# Establishment of a duplex TaqMan-based real time RT-PCR assay for simultaneous detection of BRSV and BVDV

**DOI:** 10.3389/fvets.2024.1473408

**Published:** 2024-11-04

**Authors:** Fuxing Hao, Jinping Fu, Jun Chen, Daoxian Zhu, Bingyan Cai, Yuxin Li, Chuanmin Liu

**Affiliations:** ^1^Jiangsu Agri-Animal Husbandry Vocational College, Taizhou, China; ^2^Institute of Veterinary Medicine, Jiangsu Academy of Agricultural Sciences, Nanjing, China

**Keywords:** bovine respiratory disease complex (BRDC), bovine respiratory syncytial virus (BRSV), bovine viral diarrhea virus (BVDV), TaqMan, duplex real-time RT-PCR

## Abstract

Bovine respiratory disease complex (BRDC) represents a global acute respiratory condition that imposes substantial economic burdens on the cattle industry due to its high morbidity and mortality rates. Various factors contribute to the development of BRDC, including pathogen infections, environmental stresses, weaning of calves, and herd relocation. Viral pathogens, notably bovine respiratory syncytial virus (BRSV) and bovine viral diarrhea virus (BVDV), play a critical role in the etiology of BRDC, with single or combined viral infections being particularly clinically significant. In this study, we developed a duplex TaqMan-based real-time RT-PCR assay targeting the conserved regions of the F gene of BRSV and the 5′ UTR sequence of BVDV. The limits of detection for BRSV and BVDV were 6.83 copies/μL and 5.24 copies/μL, respectively. Our validation data suggest the assay has excellent sensitivity, specificity and reproducibility. Testing of clinical samples revealed prevalence of BRSV and BVDV in local farms in Jiangsu Province, China. This study provides an efficient diagnostic tool for the epidemiological investigation of BRDC.

## 1 Introduction

Bovine respiratory disease complex (BRDC) is a systemic respiratory ailment in cattle, characterized by symptoms such as fever, nasal discharge, lethargy, dyspnea, and coughing (1). As a multifactorial and multi-etiologic syndrome, BRDC can be exacerbated by complex interactions among host factors, pathogen exposure, and environmental factors (2). Environmental conditions like tough weather, weaning, transportation, and overcrowding negatively impact both immune and non-immune defense mechanisms in cattle (3, 4). These circumstances can lead to an overall suppression of the host’s immune system, facilitating bacterial colonization in the lower respiratory tract, particularly when viral infections are present (5).

Currently, several viral pathogens, including BRSV, BVDV, IBR, bovine coronavirus (BCoV), and bovine parainfluenza virus type 3 (BPIV-3), have been identified as key contributors to the development of BRDC, with single or combined viral infections being particularly clinically significant (6, 7). Among these, BRSV and BVDV are of particular concern due to their high prevalence and significant role in causing severe respiratory illness (7–10). In China, the epidemiological situation for BRSV and BVDV underscores the importance of accurate and timely diagnosis, given that these viruses are endemic and contribute to substantial economic losses within the livestock industry (7–10).

Early and efficient diagnosis of BRSV and BVDV is crucial for controlling the spread of BRDC and mitigating economic impacts. Traditional diagnostic methods, such as virus isolation, enzyme-linked immunosorbent assay (ELISA), hemagglutination inhibition test, serum neutralization test, and SYBR Green PCR, while valuable, have limitations in terms of sensitivity, specificity, and operational complexity (11). The fluorescent quantitative PCR (qPCR) assay, on the other hand, offers a robust alternative with its ease of operation, superior sensitivity and specificity, and the capability for multiplex detection. These features make qPCR especially advantageous for rapid and accurate identification of multiple pathogens in a single reaction, thereby streamlining the diagnostic process and enabling more effective management of BRDC outbreaks.

## 2 Materials and methods

### 2.1 Viral strains and nucleic acid

Viral nucleic acids of BCoV (GenBank No. MZ603735), BPIV3 (GenBank No. MH552577), bovine rotavirus (BoRV, GenBank No. JN790188), bovine astrovirus (BoAstV, GenBank No. NC_024297), and BVDV (GenBank No. KF501393), were kept by the Institute of Veterinary Medicine, Jiangsu Academy of Agricultural Sciences.

### 2.2 Reagents and instruments

Nucleic acid extraction was conducted using the AxyPrep Body Fluid Viral DNA/RNA Miniprep Kit (AxyGen, Union City, CA, United States). RT-PCR was performed using One Step PrimeScript^™^ RT-PCR Kit (Takara Bio, Beijing, China). Primers and probes were diluted in TE buffer (Solarbio, Beijing, China). PCR amplification was carried out on an ABI StepOne Plus^™^ Real-Time PCR System (Thermo Fisher Scientific, Waltham, MA, United States).

### 2.3 Primers and probes

All available sequences of BRSV and BVDV were retrieved from GenBank for alignment using DNAMAN. Highly conserved regions from the BRSV gF gene and the BVDV 5′ UTR sequence were selected for primer and probe design (Beacon Designer 7.0 software). Sequences of primers and probes are listed in Table 1.

**Table 1:** Primers and probes designed for the duplex RT-PCR assay.

Virus	Primer/Probe	Sequence (5′–3′)	Position	Size (bp)
BRSV	BRSV-F-F1	TGCCTATAACTAATGACCAA	6,367–6,386	99
	BRSV-F-R2	TGACCTCTTCTTTGACAA	6,448–6,465	
	BRSV-F-Pr	VIC-CATAATGGAATAACTCTGTTGCCTGAC-BHQ1	6,417–6,443	
BVDV	BVDV-F	CTAGCCATGCCCTTAGTA	55–72	95
	BVDV-R	GACGACTACCCTGTACTC	132–149	
	BVDV-Pr	FAM-CTTCAGCCATCCAACGAACTCACCA-BHQ1	103–127	

### 2.4 Nucleic acid extraction

Nucleic acid extraction from cell cultures or clinical samples was performed using the Axygen Body Fluid Viral DNA/RNA Miniprep Kit, following the manufacturer’s protocol. In summary, 1 mL of 1x PBS was added to a microcentrifuge tube containing either a nasal swab or 0.5 g of a fecal sample and thoroughly mixed. The mixture was then centrifuged at 3,000 rpm for 3 min, after which 200 μL of the supernatant was used for the extraction process. This supernatant was combined with an equal volume of lysis buffer and incubated at room temperature for 5 min. Subsequently, 75 μL of V-N buffer was added to the mixture, which was then subjected to another round of centrifugation to collect the supernatant for nucleic acid isolation. The purified nucleic acid was finally eluted in 50 μL of elution buffer and stored at −80°C for subsequent analyses.

### 2.5 Standard plasmid construction

Recombinant plasmids pUC57-BRSV-F and pUC57-BVDV-5′ UTR, which contain the target fragments of BRSV and BVDV respectively, were synthesized as standard plasmids by General Biosystems Corp. Ltd. (Anhui, China).

### 2.6 Optimization of the singleplex RT-PCR assay

The reaction system was set up as recommended by the One Step PrimeScript^™^ RT-PCR Kit manual: 10 μL of 2 × One-Step RT-PCR buffer III, 0.4 μL of Ex Taq HS (5 U/μL), 0.4 μL of PrimeScript RT Enzyme Mix II, 0.8 μL of each primer set (10 μM), 0.8 μL of probe (10 μM), 0.4 μL of ROX (50×), 2 μL of nucleic acid template, and 5.2 μL of RNase free water. The total volume for each reaction is 20 μL. The reaction parameters are as follows: reverse transcription reaction: 42°C for 5 min, followed by 95°C for 10 s, 1 cycle; PCR program: 95°C for 5 s, 60°C for 30 s, 40 cycles; fluorescence signals were captured during annealing process. Six gradients of annealing temperature were set for optimization (50°C, 52°C, 54°C, 56°C, 58°C, 60°C).

### 2.7 Optimization of the duplex RT-PCR assay

In comparison with the singleplex RT-PCR, working concentrations for primers and probes were further optimized using the matrix method to screen for the optimal reaction system.

### 2.8 Specificity test of the duplex RT-PCR assay

To assess assay specificity, *in silico* analysis was performed with an online tool named Primer-BLAST.^1^ Furthermore, nucleic acids of BCoV, BPIV3, BoRV, BoAstV, BVDV viral strains were extracted and tested with the duplex RT-PCR assay.

### 2.9 Sensitivity test of the duplex RT-PCR assay

To assess assay sensitivity, 10-fold serial dilutions of 1:1 mixture of standard plasmids containing BRSV and BVDV targets were carefully tested in triplicate. The copy number for each dilution was calculated with an online software based on plasmid size and nucleic acid concentration.^2^ Limit of detection (LOD) as well as PCR amplification efficiency were determined based on standard curves generated.

### 2.10 Repeatability test of the duplex RT-PCR assay

To verify repeatability and reproducibility of the established duplex RT-PCR assay, both intra-assay and inter-assay tests were performed. Plasmid mixtures with three different concentrations (1.11 × 10^4^ copies/μL, 1.11 × 10^5^ copies/μL, and 1.11 × 10^6^ copies/μL) were prepared as samples. For the intra-assay, each sample was tested in triplicate. For the inter-assay, triplicate tests for all three samples were performed on three different dates. The coefficient of variation (CV) was calculated for each set of triplicates by dividing the standard deviation (SD) by the mean of the measurements, and then multiplying by 100 to express it as a percentage. Statistical analysis included the use of an analysis of variance (ANOVA) to assess the significance of any observed variations among the replicates and across different testing days.

### 2.11 Clinical samples collection

Twenty-three bovine fecal samples and 50 bovine nasal swabs were randomly collected from pastures and dairy farms in Jiangsu Province, respectively. All samples were preserved by the Institute of Veterinary Medicine, Jiangsu Academy of Agricultural Sciences. All clinical samples were tested using both the duplex and singleplex RT-PCR assays established in this study.

## 3 Results

### 3.1 Development and optimization of the duplex real-time RT-PCR assay

The optimized annealing temperature for singleplex real-time PCR is 50°C. After optimization with the matrix method, the final concentrations of primers and probes are 0.6 μmol/L and 0.4 μmol/L for BRSV, 0.2 μmol/L and 0.2 μmol/L for BVDV, respectively (Table 2). The optimized reaction procedure was as follows: reverse transcription reaction: 42°C for 5 min, followed by 95°C for 10 s, 1 cycle; PCR program: 95°C for 5 s, 50°C for 30 s (annealing), 40 cycles, with fluorescence signals collected during the annealing step.

**Table 2:** Optimal reaction system for the duplex RT-PCR assay.

Reagents	Volume (μL)	Final concentration (μM)
2 × One-Step RT-PCR buffer III	10	
BRSV primer set (10 μM)	1.2	0.6
BVDV primer set (10 μM)	0.4	0.2
BRSV probe	0.8	0.4
BVDV probe	0.4	0.2
ROX (50×)	0.4	
Ex Taq HS (5 U/μL)	0.4	
PrimeScript RT Enzyme Mix II	0.4	
Nucleic acid template	2	
RNase free water	Up to 20	

The judgement criteria are as follows: the test is valid only when both the negative and positive controls work. If the sample has no fluorescence amplification curve, it is considered negative; if the sample has a Ct value less than 35 with a fluorescence amplification curve, it is considered positive; if the sample has a Ct value between 35 and 40 with a fluorescence amplification curve, it is considered suspect and needs to be re-extracted and re-tested for confirmation. If the Ct value is again between 35 and 40 with an amplification curve, the sample is considered positive.

### 3.2 Sensitivity and standard curves of the duplex real-time RT-PCR assay

**Figure 1:**
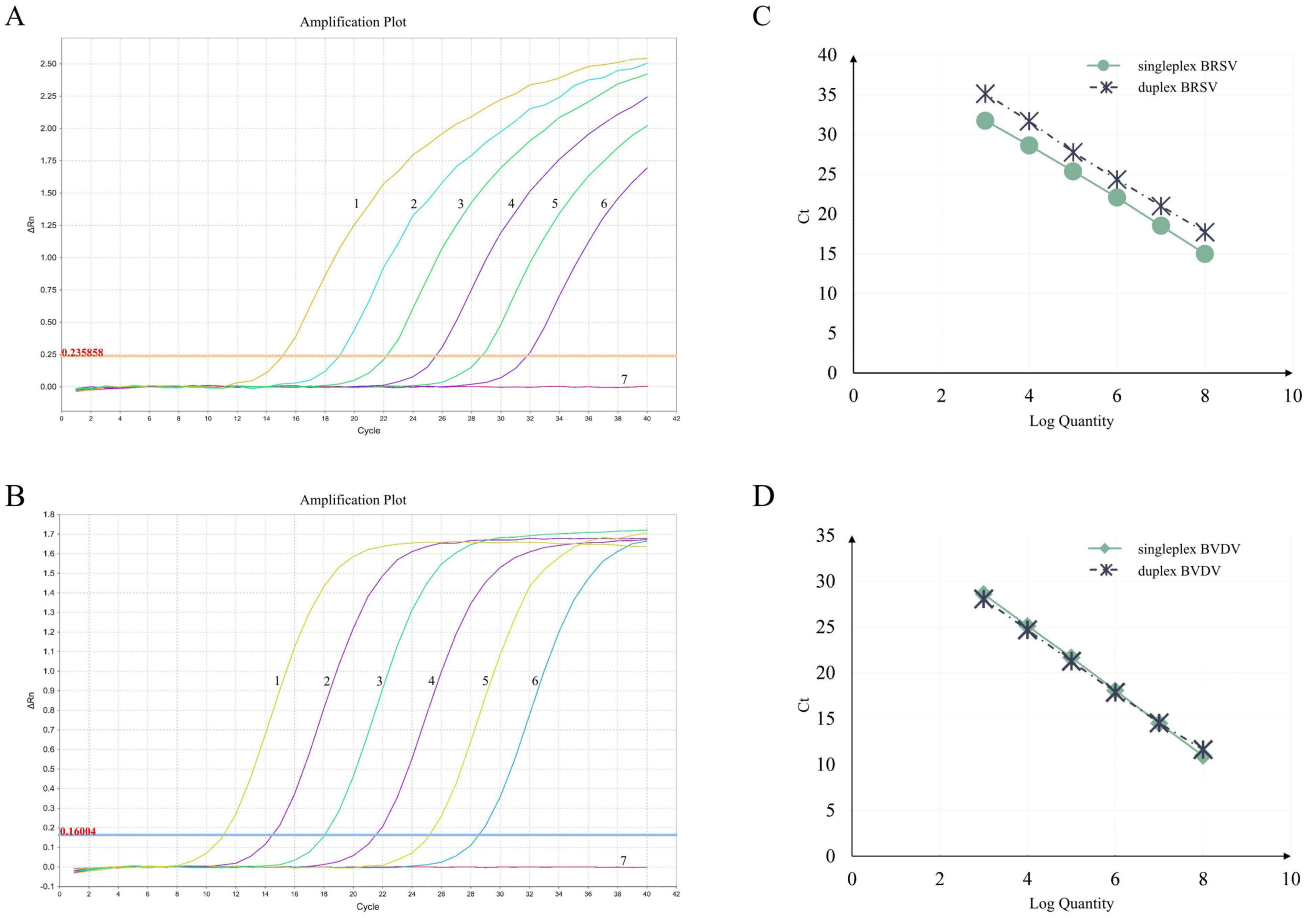
Amplification curves and standard curves of the duplex RT-PCR assay. The amplification curves for BRSV **(A)** and BVDV **(B)** were generated using recombinant standard plasmids pUC57-BRSV-F and pUC57-BVDV-5′ UTR, respectively. The standard curves **(C,D)** were generated from the amplification curves. In **(A,B)**, the plasmid concentrations for curves 1 to 6 ranged from 6.83 × 10^8^ to 6.83 × 10^3^ copies/μL and from 5.24 × 10^8^ to 5.24 × 10^3^ copies/μL for BRSV and BVDV, respectively. Curve 7 represents the negative control.

To assess the sensitivity of the assay, 10-fold serial dilutions of 1:1 mixture of standard plasmids containing BRSV and BVDV targets (ranging from 6.83 × 10^8^ to 6.83 × 10^0^ copies/μL and from 5.24 × 10^8^ to 5.24 × 10^0^ copies/μL for BRSV and BVDV, respectively) were carefully tested in triplicate. The standard curves for both singleplex and duplex assays was generated by plotting the Ct values against logarithm of the template quantities (Figure 1). PCR amplification efficiencies were all between 90 and 100.30% (90.02, 93.17% for BRSV singleplex and duplex assays, respectively, 94.71, 100.30% for BVDV singleplex and duplex assays, respectively). At the same time, correlation coefficients (*R*^2^) were all above 0.999 (1, 0.9992 for BRSV singleplex and duplex assays, respectively, 0.9996, 0.9993 for BVDV singleplex and duplex assays, respectively). These data indicate no inhibition of PCR sensitivity in the multiplex assay compared to the singleplex assays. The limit of detection for both BRSV and BVDV targets was less than 10 copies (Table 3).

**Table 3:** Parameters of the standard curves.

Virus	Amplification efficiency (%)		*R* ^2^		Limit of detection (LOD) (copies)	
	Singleplex	Duplex	Singleplex	Duplex	Singleplex	Duplex
BRSV	90.02%	93.17%	1	0.9992	6.83	6.83
BVDV	94.71%	100.30%	0.9996	0.9993	5.24	5.24

### 3.3 Specificity of the duplex real-time RT-PCR assay

**Figure 2:**
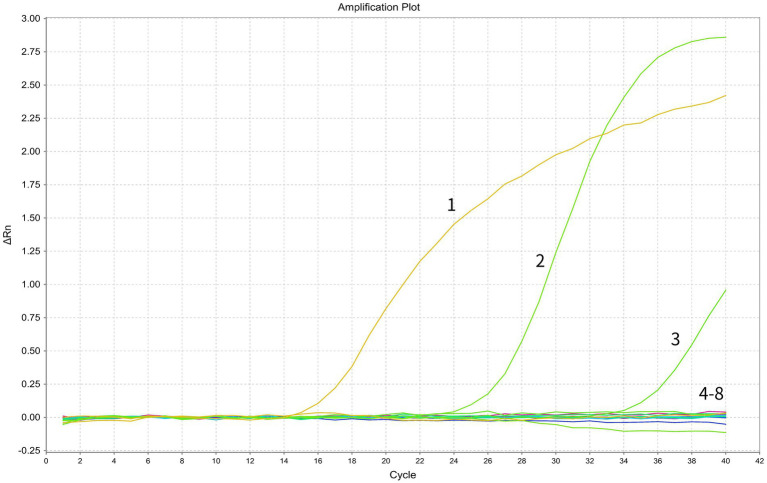
Specificity test of the duplex RT-PCR assay. In the displayed amplification plot, the *x*-axis represents the cycle number, while the *y*-axis represents the ΔRn value. Details of the amplification curves are as follows: 1, BRSV standard plasmid; 2, BVDV standard plasmid; 3, BVDV viral nucleic acid; 4–7, viral nucleic acids of BCoV, BPIV3, BoRV, and BoAstV; 8, negative control.

The specificity of the primers and probes was first assessed by *in silico* analysis using the NCBI primer-BLAST function, which determined a unique target for each set of the assay. Subsequently, the assay specificity was evaluated using specific viral targets (standard plasmids containing BRSV or BVDV, and nucleic acid of a BVDV viral strain) and non-target viral pathogens (nucleic acids of multiple bovine viral strains, including BCoV, BPIV3, BoRV and BoAstV). The assay specifically detected BRSV and BVDV while no positive signals were generated from any of the other non-target pathogens, indicating good specificity of the assay (Figure 2).

### 3.4 Repeatability and reproducibility of the duplex real-time RT-PCR assay

The intra-assay and inter-assay variations of Ct values were carefully determined. The analysis revealed that the coefficients of variation (CV) for both BRSV and BVDV are below 0.02% (Table 4), which suggests that the developed duplex real-time RT-PCR assay has high repeatability and stability.

**Table 4:** Repeatability and reproducibility of the duplex RT-PCR assay.

Virus standards	Concentration (copies/μL)	Intra-assay			Inter-assay		
		Ct (mean)	SD	CV (%)	Ct (mean)	SD	CV (%)
BRSV	1.11 × 10^6^	23.72	0.13	0.01	24.00	0.31	0.01
	1.11 × 10^5^	27.12	0.01	0.00	27.41	0.32	0.01
	1.11 × 10^4^	30.61	0.05	0.00	31.15	0.53	0.02
BVDV	1.11 × 10^6^	18.29	0.02	0.00	18.20	0.29	0.02
	1.11 × 10^5^	21.79	0.03	0.00	21.62	0.30	0.01
	1.11 × 10^4^	25.35	0.06	0.00	25.10	0.36	0.01

### 3.5 Application and detection of the duplex real-time RT-PCR assay

A total of 73 clinical samples were tested with the duplex real-time RT-PCR assay. The positive rates for BRSV and BVDV were 5.48% (4/73) and 8.22% (6/73), respectively. No co-infections were detected. To confirm the data, all clinical samples were also tested with the singleplex real-time RT-PCR assay. The results showed a 100% match.

## 4 Discussion

BRDC is a significant and costly issue in the global cattle industry, due to its complex etiology and the involvement of multiple pathogens (12, 13). Among these, BRSV and BVDV are frequently identified in co-infection, which can lead to increased morbidity and mortality rates (7–10).

BRSV, an enveloped, single-stranded, negative-sense RNA virus, is known for its high morbidity, particularly when combined with other viral or bacterial infections (14). A recent study in China reported a 18.65% positivity rate for BRSV across 23 farms in 11 provinces (9), while another study in Sichuan Province found an even higher average positive rate of 64.57% among yaks (15). BVDV, on the other hand, is an enveloped, single-stranded, positive-sense RNA virus that can be transmitted both vertically and horizontally (16). It has been shown to have a high seroprevalence in East China, with a 77.8% positive rate for BVDV antibodies in bulk tank milk samples, and a lower, but still concerning, 1.86% antigen-positive rate in individual cows. In our study, we observed a positive rate of 8.22% for BRSV and 5.48% for BVDV, without any cases of mixed infection (17).

The multifactorial nature of BRDC makes its management challenging, underscoring the need for rapid and accurate diagnostic tools. Conventional methods, such as serological tests and single-target PCR, have limitations, including cross-reactivity, low sensitivity, and the inability to detect multiple pathogens simultaneously. The development of a duplex TaqMan-based fluorescence quantitative RT-PCR assay for the simultaneous detection of BRSV and BVDV addresses some of these shortcomings. This method offers several advantages over traditional approaches, including: (1) high specificity and sensitivity: the use of specific primers and probes allows for the precise identification of target viruses, reducing the likelihood of false positives or negatives; (2) reproducibility and repeatability: standardized conditions ensure consistent results, which is critical for both routine diagnostics and epidemiological studies; (3) economic and systematic convenience: by detecting two viruses in a single reaction, this assay reduces the cost and time associated with running separate tests, making it more practical for large-scale screening; (4) multiplexing capability: the ability to test for multiple targets simultaneously increases the efficiency of surveillance and monitoring efforts, especially in areas where co-infections are common.

However, the design and validation of such a multiplex assay are not without challenges. One of the key difficulties lies in optimizing the amplification conditions to achieve balanced and efficient detection of all targets. This involves carefully selecting primers and probes that do not cross-react and have similar annealing temperatures, as well as ensuring that the PCR conditions are optimized for all targets. Additionally, the presence of multiple targets in a single reaction can sometimes lead to competition for reagents, which can affect the sensitivity and specificity of the assay. Another significant challenge is ensuring that the assay maintains its performance characteristics across different laboratories and sample types. This requires rigorous standardization and validation processes. Standardization involves developing detailed protocols and guidelines that can be consistently followed by different laboratories. Validation, on the other hand, includes extensive testing to confirm the assay’s accuracy, precision, sensitivity, and specificity. This often involves the use of well-characterized reference materials and the inclusion of positive and negative controls to monitor the performance of the assay over time.

In a summary, a duplex TaqMan-based fluorescence quantitative RT-PCR assay for the simultaneous detection of two bovine respiratory viruses, BRSV and BVDV, was well established. Its high specificity, sensitivity, and reproducibility, coupled with the economic and systematic benefits, make it a valuable tool for both clinical diagnostics and epidemiological investigations. By overcoming the limitations of conventional assays, this method provides a robust platform for the improved management and control of BRDC.

## Data Availability

The raw data supporting the conclusions of this article will be made available by the authors, without undue reservation.

## Footnotes

^1^ https://www.ncbi.nlm.nih.gov/tools/primer-blast/

^2^ https://www.technologynetworks.com/tn/tools/copynumbercalculator
